# Experimental solubility and thermodynamic modeling of *empagliflozin* in supercritical carbon dioxide

**DOI:** 10.1038/s41598-022-12769-2

**Published:** 2022-05-30

**Authors:** Gholamhossein Sodeifian, Chandrasekhar Garlapati, Fariba Razmimanesh, Hassan Nateghi

**Affiliations:** 1 Department of Chemical Engineering, Faculty of Engineering, University of Kashan, Kashan, 87317-53153 Iran; 2 Laboratory of Supercriritcal Fluids and Nanotechnology, University of Kashan, Kashan, 87317-53153 Iran; 3 Modeling and Simulation Centre, Faculty of Engineering, University of Kashan, Kashan, 87317-53153 Iran; 4 Department of Chemical Engineering, Puducherry Technological University, Puducherry, 605014 India

**Keywords:** Chemical engineering, Engineering, Chemistry, Chemical engineering

## Abstract

The solubility of *empagliflozin* in supercritical carbon dioxide was measured at temperatures (308 to 338 K) and pressures (12 to 27 MPa), for the first time. The measured solubility in terms of mole faction ranged from 5.14 × 10^–6^ to 25.9 × 10^–6^. The cross over region was observed at 16.5 MPa. A new solubility model was derived to correlate the solubility data using solid–liquid equilibrium criteria combined with Wilson activity coefficient model at infinite dilution for the activity coefficient. The proposed model correlated the data with average absolute relative deviation (AARD) and Akaike’s information criterion (AIC_c_), 7.22% and − 637.24, respectively. Further, the measured data was also correlated with 11 existing (three, five and six parameters empirical and semi-empirical) models and also with Redlich-Kwong equation of state (RKEoS) along with Kwak-Mansoori mixing rules (KMmr) model. Among density-based models, Bian et al., model was the best and corresponding AARD% was calculated 5.1. The RKEoS + KMmr was observed to correlate the data with 8.07% (correspond AIC_c_ is − 635.79). Finally, total, sublimation and solvation enthalpies of *empagliflozin* were calculated.

## Introduction

**Figure 1:**
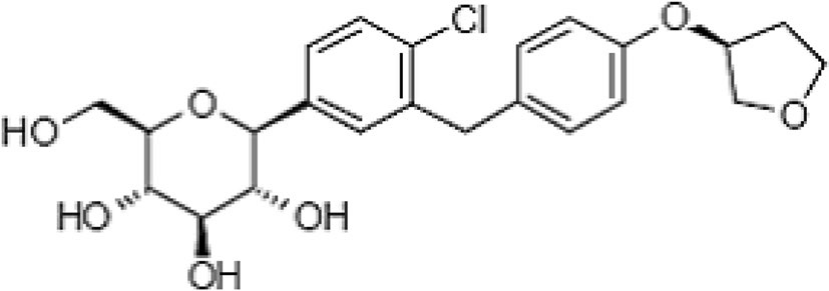
*Empagliflozin* chemical structure.

Supercritical carbon dioxide (ScCO_2_) is a fluid above its critical point. It has physical properties (density, diffusivity, viscosity and surface tension) intermediate to that of gas and liquid^1,2^. ScCO_2_ has been used as a solvent in various process applications, because it has gas like diffusivity and liquid like density with low viscosity and surface tension^1,3–5^. The major applications are in drug particle micronization, food processing, textile dyeing, ceramic coating, extraction and many more^4,6–12^. Although, several supercritical fluids are utilized as solvent in process industry, ScCO_2_ is the most desirable solvent^8,13–17^. In general, phase equilibrium information is necessary to implement supercritical fluid technology (SFT)^6,7,9^. The solubility is the basic information for the design and development of SFT. In literature, solubility of many drug solids in ScCO_2_ is readily available^18–30^, however, the solubility of *empagliflozin* has not been reported, therefore in this work for the first time, its solubility in ScCO_2_ has been measured. This data may be used in the particle micronization process using ScCO_2_. The molecular formula of *empagliflozin* is C_23_H_27_ ClO_7_ and its molecular weight is 450.91. The chemical structure is shown in Fig. 1.

*Empagliflozin* is an inhibitor of sodium-glucose co-transporter-2 (SGLT2), the transporters primarily responsible for the re-absorption of glucose in the kidney. Further, it is useful in reducing the risk of cardiovascular death in adults with type 2 diabetes mellitus and cardiovascular disease^31^. Sufficient drug dosage is very essential for those treatments and this is achieved through a proper particle size. Therefore, the present study is quite useful in particle micronization using ScCO_2_. Solubility measurement at each desired condition is very cumbersome and hence, there is a great need to develop a model that correlates/predicts the solubility^32^. Recent developments such as machine learning methods may be considered with the improvement of artificial intelligence prediction methods for the data correlation^33–35^. However, in general, the solubility models are classified into five types; however, only three are user friendly, and they are equation of state, density-based and mathematical models^36^. Directly or indirectly all of them are derived based on thermodynamic frame work. The derived models make use of the basic concepts related to phase equilibrium criteria (solid–gas or solid–liquid), solvent–solute association theory, dilute solution theory, solution theory and Wilson model or any other model^37^. In fact, most of the literature models correlate the solubility of the solid solutes in ScCO_2_ quite well. A solid–gas equilibrium models need the critical properties and vapour pressure of the solute, while these properties are rarely available in literature, due to this, the group contribution methods are commonly used^38^. On the other hand, the solid–liquid equilibrium (SLE) criterion requires an appropriate model for activity coefficient calculation. A recent study reveals that SLE model in combination with Van Laar activity coefficient model can be a simple approach in model development, but this method resulted in an implicit expression in terms of mole fraction^38,39^. Therefore, there is a need to develop an explicit solubility model and hence, this task is taken up in this work.

The main motives of this study were in two levels. In the first level, *empagliflozin* solubility in ScCO_2_ was determined and in the second level, a new explicit solubility model was developed based on solid–liquid equilibrium criterion in combination with Wilson activity coefficient model for the activity coefficient calculation.

## Experimental

### Materials

Gaseous CO_2_ (purity > 99.9%) was obtained from Fadak company, Kashan (Iran), *empagliflozin* (CAS Number: 864070-44-0, purity > 99%) was purchased from Amin Pharma company, and dimethyl sulfoxide (DMSO, CAS No. 67-68-5, purity > 99%) was provided from Sigma Aldrich company. Table 1 indicates all the information about the chemicals utilized in this work.

**Table 1:** Some physicochemical properties of the used materials.

Compound	Formula	M_W_ (g/mol)	T_m_ (K)	λ_max_ (nm)	CAS number	Minimum purity by supplier
Empagliflozin	C_23_H_27_ClO_7_	450.9	426.1	276	864070-44-0	99%
Carbon dioxide	CO_2_	44.01			124-38-9	99.99%
DMSO	C_2_H_6_OS	78.13			67-68-5	99%

### Experiment details

**Figure 2:**
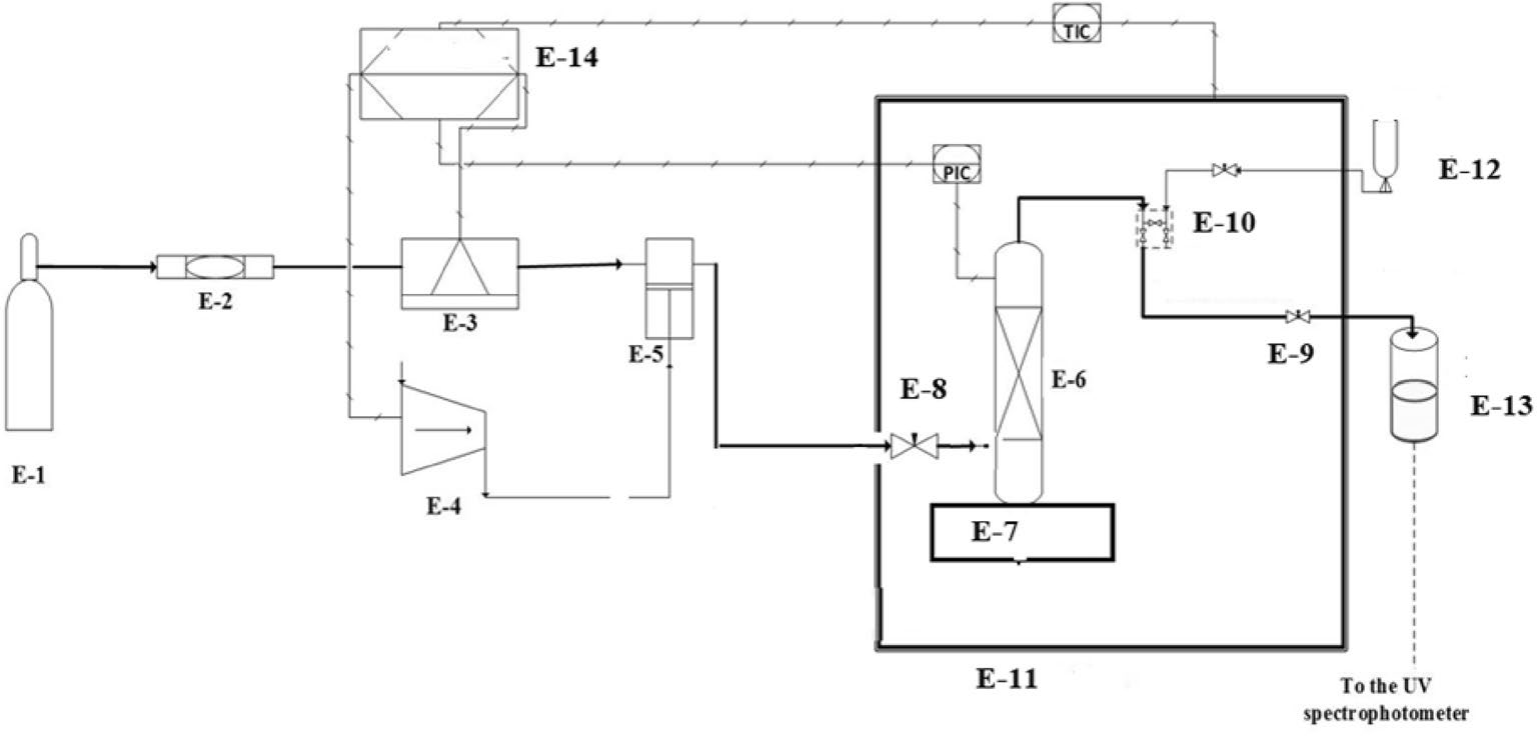
Experimental setup for solubility measurement, E1—CO_2_ cylinder; E-2—Filter; E-3—Refrigerator unit; E-4—Air compressor; E-5—High pressure pump; E-6—Equilibrium cell; E-7—Magnetic stirrer; E-8—Needle valve; E-9—Back-pressure valve; E-10—Six-port, two position valve; E-11—Oven; E-12—Syringe; E13—Collection vial; E-14—Control panel.

The detailed discussion of the solubility apparatus and equilibrium cell has been presented in our earlier studies (Fig. 2)^19,25,40,41^. However, a brief description about the apparatus is presented in this section. This method may be classified as an isobaric-isothermal method^42^. Each measurement was carried out with high precision and temperatures and pressures were controlled within ± 0.1 K and ± 0.1 MPa, respectively. For all measurement, 1 g of *empagliflozin* drug was used. As mentioned in our previous works, the equilibrium was observed within 60 min. After equilibrium, 600 µL saturated ScCO_2_ sample was collected via 2-status 6-way port valve in a DMSO preloaded vial. After discharging 600 µL saturated ScCO_2_, the port valve was washed with 1 ml DMSO. Thus, the total saturation solution was 5 ml. Each measurement was repeated thrice and their average values were reported. Mole fraction is obtained as follows:

1 $$ y_{2} = \frac{{n_{drug} }}{{n_{drug} + n_{{CO_{2} }} }} $$

where ${n}_{\text{solute}}$ is the moles number of the drug, and ${n}_{{\text{CO}}_{2}}$ is the moles number of CO_2_ in the sampling loop.

Further, the above quantities are given as:

2 $${n}_{\text{solute}}=\frac{{C}_{s}\cdot {V}_{s}}{{M}_{s}}$$

3 $${n}_{{\text{CO}}_{2}}=\frac{{V}_{1}\cdot \rho }{{M}_{{\text{CO}}_{2}}}$$

where ${C}_{\text{s}}$ is the drug concentration in saturated sample vial in g/L. The volume of the sampling loop and vial collection are V_1_(L) = 600 $\times$ 10^–6^ m^3^ and V_s_(L) = 5 $\times$ 10^–3^ m^3^, respectively. The $M_{s}$ and $M_{{\text{CO}_{2} }}$ are the molecular weight of drug and CO_2_, respectively. Solubility is also described as

4 $$ S = \frac{{C_{S} V_{s} }}{{V_{1} }} $$

The relation between S and $y_{2}$ is

5 $$ S = \frac{{\rho M_{s} }}{{M_{{\text{CO}_{2} }} }}\frac{{y_{2} }}{{1 - y_{2} }} $$

A UV–Visible spectrophotometer (Model UNICO-4802) and DMSO solvent were used for the measurement of *empagliflozin* solubility. The samples were analyzed at 276 nm.

## Existing and new models and their correlations

In this section, the details of various solubility models are presented along with a new explicit solubility model.

### Existing models

#### Alwi–Garlapati model (three parameters model)^43^

It is one of the latest models for the solubility correlation. It is mathematically explained as

6 $$ y_{2} = \frac{1}{{\rho_{r} T_{r} }}\exp \left( {A_{1} + \frac{{B_{1} }}{{T_{r} }} + C_{1} \rho_{r} } \right) $$

where $A_{1} - \;C_{1}$ are model constants.

#### Bartle et al., model (three parameters model)^44^

It is an empirical model and mathematically stated as:

7 $$ \ln \left( {\frac{{y_{2} P}}{{P_{ref} }}} \right) = A_{2} + \frac{{B_{2} }}{T} + C_{2} \left( {\rho - \rho_{ref} } \right) $$

where $A_{2} - C_{2}$ are model constants. From parameter $B_{2}$, one can estimate sublimation enthalpy using the relation, $\Delta_{sub} H = - B_{2} R$, in which *R* is universal gas constant.

#### Bian et al., model (five parameters model)^45^

It is an empirical model and mathematically formulated as:

8 $$ y_{2} = \rho_{1}^{{\left( {A_{3} + B_{3} \rho_{1} } \right)}} \exp \left( {{{C_{3} } /T} + D_{3} {{\rho_{1} } / T} + E_{3} } \right) $$

where $A_{3} - E_{3}$ are model constants.

#### Chrastil model (three parameters model)^46^

It is a semi-empirical model and mathematically stated as:

9 $$ c_{2} = \rho_{1}^{\kappa } \exp \left( {A_{4} + \frac{{B_{4} }}{T}} \right) $$

where $\kappa ,A_{4} \;{\text{and}}\;B_{4}$ are model constants.

In terms of mole fraction, it is written as^47^:

9a $$ y_{2} = \frac{{\left( {\rho_{1} } \right)^{\kappa - 1} \exp \left( {A_{4} + \frac{{B_{4} }}{T}} \right)}}{{\left[ {1 + \left( {\rho_{1} } \right)^{\kappa - 1} \exp \left( {A_{4} + \frac{{B_{4} }}{T}} \right)} \right]}} $$

#### Garlapati–Madras model (five parameters model)^48^

It is a mathematical model and mathematically formulated as

10 $$ \ln \left( {y_{2} } \right) = A_{5} + (B_{5} + C_{5} \rho_{1} )\ln \left( {\rho_{1} } \right) + \frac{{D_{5} }}{T} + E_{5} \ln \left( {\rho_{1} T} \right) $$

where $A_{5} - E_{5}$ are model constants.

#### Kumar–Johnstone model (three parameters model)^49^

It is a semi empirical model and mathematically described as:

11 $$ \ln \left( {y_{2} } \right) = A_{6} + B_{6} \rho + {C_{6}} /T $$

where $A_{6} - C_{6}$ are model constants.

#### Mahesh–Garlapati model (three parameters model)^39^

It is one of the latest models. It is based on degree of freedom and mathematically stated as:

12 $$ \ln \left( {y_{2} } \right) = A_{7} + B_{7} \rho_{r} T_{r} + C_{7} \rho_{r} T_{r}^{3} $$

where $A_{7} - C_{7}$ are model constants.

#### Mendez–Teja model (three parameters model)^50^

It is a semi-empirical model and mathematically explained as:

13 $$ T\ln \left( {y_{2} P} \right) = A_{8} + B_{8} \rho + C_{8} T $$

where $A_{8} - C_{8}$ are model constants.

#### Sodefian et al., model (six parameters model)^40^

It is a mathematical model and stated as:

14 $$ \ln \left( {y_{2} } \right) = A_{9} + \frac{{B_{9} P^{2} }}{T} + C_{9} \ln \left( {\rho_{1} T} \right) + D_{9} \left( {\rho_{1} \ln \left( {\rho_{1} } \right)} \right) + E_{9} P\ln \left( T \right) + F_{9} \frac{{\ln \left( {\rho_{1} } \right)}}{T} $$

where $A_{9} - F_{9}$ are model constants.

#### Reformulated Chrastil model (three parameters model)^47,51^

It is a semi-empirical model and mathematically explained as:

15 $$ y_{2} = \left( {\frac{{RT\rho_{1} }}{{M_{CF} f^{ \cdot } }}} \right)^{{\kappa^{\prime} - 1}} \exp \left( {A_{11} + \frac{{B_{11} }}{T}} \right) $$

where $\kappa^{\prime},A_{10} \;{\text{and}}\;B_{10}$ are model constants.

#### Tippana–Garlapati model (six parameters model)^52^

It is a degree of freedom model and mathematically stated as:

16 $$ y_{2} = \left( {A_{12} + B_{11} P_{r} + C_{11} P_{r}^{2} } \right)T_{r}^{2} + (D_{11} + E_{11} P_{r} + F_{11} P_{r}^{2} ) $$

where $A_{11} - F_{11}$ are model constants.

### New model

According to solid–liquid phase equilibrium criteria, the fugacity of the solute in the solid phase and liquid phase is equal at equilibrium. The liquid phase is considered as an expanded liquid phase of ScCO_2_. At equilibrium, the solubility may be expressed as^53–57^

17 $$ y_{2} = \frac{1}{{\gamma_{2}^{\infty } }}\frac{{f_{2}^{S} }}{{f_{2}^{L} }} $$

where $\gamma_{2}^{\infty }$ is drug activity coefficient at infinitesimal dilution in ScCO_2_ and $f_{2}^{S}$ and $f_{2}^{L}$ are fugacities of drug in the solid and ScCO_2_ phases, respectively. The ${f_{2}^{S} }/{f_{2}^{L} }$ ratio may be expressed as follows:

18 $$ \ln \left( {\frac{{f_{2}^{S} }}{{f_{L}^{L} }}} \right) = \frac{{\Delta H_{2}^{m} }}{RT}\left( {\frac{T}{{T_{m} }} - 1} \right) - \int\limits_{{T_{m} }}^{T} {\frac{1}{{RT^{2} }}} \left[ {\int\limits_{{T_{m} }}^{T} {\left[ {\Delta C_{p} } \right]dT} } \right]dT $$

where,$\Delta C_{p}$ is heat capacity difference of the drug in solid phase and that of SCCO_2_ phase. The terms that include △Cp is much smaller than the term that has $\Delta H_{2}^{m}$^58^, thus leaving △Cp term yields a much simpler expression for fugacity ratio as:

19 $$ \ln \left( {\frac{{f_{2}^{S} }}{{f_{L}^{L} }}} \right) = \frac{{\Delta H_{2}^{m} }}{RT}\left( {\frac{T}{{T_{m} }} - 1} \right) $$

Combining Eq. (19) with Eq. (17) give the expression for the solubility model (Eq. (20)).

20 $$ y_{2} = \frac{1}{{\gamma_{2}^{\infty } }}\exp \left[ {\frac{{\Delta H_{2}^{m} }}{RT}\left( {\frac{T}{{T_{m} }} - 1} \right)} \right] $$

In order to use Eq. (20), the appropriate model for $\gamma_{2}^{\infty }$ is essential.

In this work, the required activity coefficient is obtained from Wilson activity coefficient model^56^ at infinite dilution and it is given by the Eq. (21).

21 $$ \gamma_{2}^{\infty } = \exp \left[ { - \ln \left( {\lambda_{21} } \right) + 1 - \lambda_{12} } \right] $$

where $\lambda_{12} = \left( {{{V_{2} }/ {V_{1} }}} \right)\exp \left( { - {{a_{12} } /{RT}}} \right)$ and $\lambda_{21} = \left( {{{V_{1} } / {V_{2} }}} \right)\exp \left( { - {{a_{21} } /{RT}}} \right)$, $V_{1}$ and $V_{2}$ are molar volumes of solvent and solute, respectively.

When $\rho_{1} = {1 / {V_{1} }}$, the final expression for the infinite dilution activity coefficient is obtained as:

22 $$ \gamma_{2}^{\infty } = \exp \left[ {1 + \ln \left( {\rho_{1} V_{2} } \right) + \frac{{a_{21} }}{RT} - \rho_{1} V_{2} \exp \left( {\frac{{ - a_{12} }}{RT}} \right)} \right] $$

The quantities $a_{12}$ and $a_{21}$ are assumed to be functions of reduced solvent density^57^, and molar volume of the solute is assumed as a constant value. In this work, $a_{12}$ and $a_{21}$ are assumed to have the following form:

23 $$ a_{12} = A\left( {\rho_{r} } \right)^{B} $$

24 $$ a_{21} = C\left( {\rho_{r} } \right)^{D} $$

Combining Eqs. (22), (23) and (24) with Eq. (20), give the following new explicit solubility model:

25 $$ y_{2} = {{\exp \left[ {\frac{{\Delta H_{2}^{m} }}{RT}\left( {\frac{T}{{T_{m} }} - 1} \right)} \right]} / {\exp \left[ {1 + \ln \left( {\rho_{1} V_{2} } \right) + \frac{{A\left( {\rho_{r} } \right)^{B} }}{RT} - \rho_{1} V_{2} \exp \left( {\frac{{ - C\left( {\rho_{r} } \right)^{D} }}{RT}} \right)} \right]}} $$

Equation (25) has four temperature independent adjustable variables namely $A$,$B$,$C$ and $D$.

### Equation of state (EoS) model

The solubility of drug *i* (solute) in ScCO_2_ (solvent) is expressed as^59–61^:

26 $$ {\text{y}}_{{\text{i}}} = \frac{{P_{i}^{S} \hat{\varphi }_{i}^{S} }}{{P\hat{\varphi }_{i}^{{ScCO_{2} }} }}\exp \left[ {\frac{{\left( {P - P_{i}^{S} } \right)V_{S} }}{RT}} \right] \, $$

where $P_{i}^{s}$ is the sublimation pressure of the pure solid at system temperature T, P is the system pressure,$V_{s}$ is the molar volume of the pure solid, *R* is the universal gas constant. The fugacity coefficient of the pure solute at saturation ($\hat{\varphi }_{i}^{S}$) is usually taken to be unity. In this work, the fugacity coefficient of the solute in the supercritical phase $\hat{\varphi }_{i}^{{ScCO_{2} }}$ is calculated using EoS along with KMmr^57^. The expression used for calculation of $\hat{\varphi }_{i}^{{ScCO_{2} }}$ is obtained from the following basic thermodynamic relation^60^:

27 $$ \ln \left( {\hat{\varphi }_{i}^{{ScCO_{2} }} } \right) = \frac{1}{RT}\int\limits_{v}^{\infty } {\left[ {\left( {\frac{\partial P}{{\partial N_{i} }}} \right)_{{T,V,N_{j} }} - \frac{RT}{v}} \right]} dv - \ln Z $$

The expression for $\hat{\varphi }_{i}^{{ScCO_{2} }}$ is

28 $$ \begin{aligned}   \ln \left( {\hat{\varphi }_{i}^{{ScCO_{2} }} } \right) & = \ln \left( {\frac{v}{{v - b}}} \right) + \left( {\frac{{2\sum {x_{j} b_{{ij}}  - b} }}{{v - b}}} \right) - \ln \left( Z \right) \hfill \\    & \quad + \left( {\frac{{a\left( {2\sum {x_{j} b_{{ij}}  - b} } \right)}}{{b^{2} RT^{{3/2}} }}} \right)\left[ {\ln \left( {\frac{{v + b}}{v}} \right) - \frac{b}{{v + b}}} \right]\left( {3\alpha ^{{1/2}} {{\left( {\sum {x_{j} a_{{ij}}^{{2/3}} b_{{ij}}^{{1/3}} } } \right)} / {b^{{1/2}} }} - \alpha ^{{2/3}} \left( {{{\sum {x_{j} b_{{ij}} } } / {b^{{3/2}} }}} \right)} \right)/bRT^{{3/2}}  \hfill \\  \end{aligned} $$

$${\text{where}}$$ $$\alpha  = \sum\limits_{i}^{n} {\sum\limits_{j}^{n} {x_{i} x_{j} a_{{ij}}^{{2/3}} } } b_{{ij}}^{{1/3}} $$

and the associated mixing rules are:

29 $$ a = \frac{{\left( {\sum\limits_{i}^{n} {\sum\limits_{j}^{n} {x_{i} x_{j} a_{{ij}}^{{2/3}} b_{{ij}}^{{1/3}} } } } \right)^{{3/2}} }}{{\left( {\sum\limits_{i}^{n} {\sum\limits_{j}^{n} {x_{i} x_{j} b_{{ij}}^{{1/2}} } } } \right)^{{1/2}} }}$$

30 $$ b = \sum\limits_{i}^{n} {\sum\limits_{j}^{n} {x_{i} x_{j} b_{ij} } } $$

31 $$ a_{ij} = \left( {1 - k_{ij} } \right)\sqrt {a_{ii} a_{jj} } $$

32 $$ b_{{ij}}  = \left( {1 - l_{{ij}} } \right)\frac{{\left( {b_{{ii}}^{{^{{1/3}} }}  + b_{{jj}}^{{1/3}} } \right)^{3} }}{8} $$

The main reason for considering RKEoS is that it has only two adjustable constants $k_{ij}$ and $l_{ij}$.

All the models (density-based, new and RKEoS models) are correlated with the following objective function^58^:

33 $$ OF = \sum\limits_{i = 1}^{N} {\frac{{\left| {y_{2i}^{\exp } - y_{2i}^{calc} } \right|}}{{y_{2i}^{\exp } }}} $$

The regression ability of a model is indicated in terms of an average absolute relative deviation percentage (AARD %).

34 $${\text{AARD }}\left( \%  \right){\text{ }} = \left( {{{100} / {N_{i} }}} \right)\sum\limits_{{i = 1}}^{N} {\frac{{\left| {y_{{2i}}^{{\exp }}  - y_{{2i}}^{{cal}} } \right|}}{{y_{{2i}}^{{\exp }} }}}$$

For the regression, fminsearch (MATLAB 2019a^®^) algorithm has been used.

## Results and discussion

**Figure 3:**
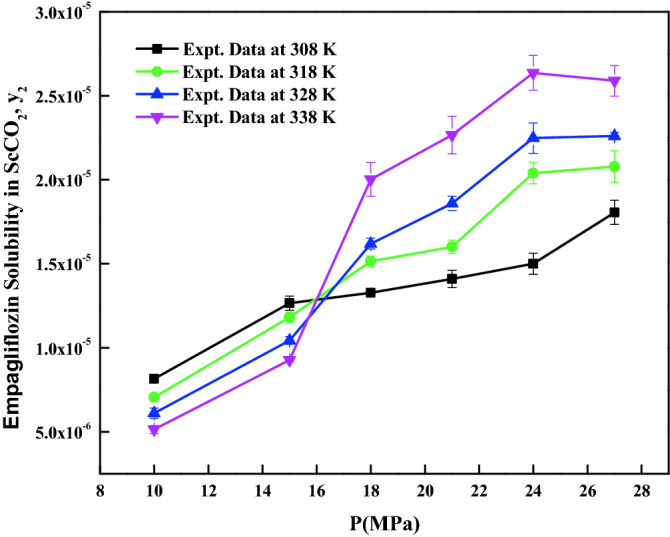
*Empagliflozin* solubility in ScCO_2_ vs. pressure.

**Figure 4:**
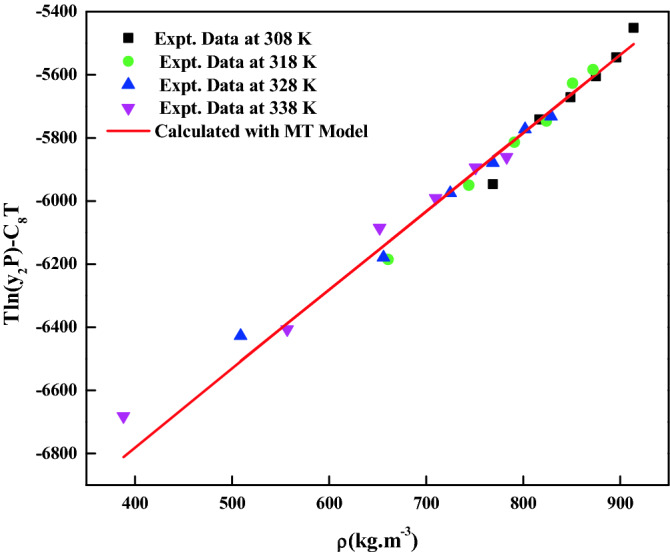
Self-consistency plot based on MT model.

Table 1 shows some physicochemical properties of the used materials. *Empagliflozin* solubility in ScCO_2_ is reported at various temperatures (T = 308 to 338 K) and pressures (P = 12 to 27 MPa). Table 2 indicates the solubility data and ScCO_2_ density. The reported ScCO_2_ density is obtained from the NIST data base. Figure 3 shows the effect of pressure on various isotherms. The cross over region is observed at 16.5 MPa. From Fig. 3, below the cross over region, solubility decreases with increase in temperature, and on the other hand, above the cross over region, the solubility increases with increase in temperature. The EoS model requires critical properties which are computed with standard group contribution methods based on the chemical structure^62–65^. The summary of the critical properties computed are shown in Table 3. Figure 4 presents the self-consistency of the measured data with MT model.

**Table 2:** Solubility of crystalline *empagliflozin* in ScCO_2_ at various temperatures and pressures.

Temperature (K)^a^	Pressure (MPa)^a^	Density of ScCO_2_ (kg/m^3^)^71^	y_2_ × 10^4^ (mole fraction)	Experimental standard deviation, S(ȳ) × (10^4^)	S (equilibrium solubility) (g/L)	Expanded uncertainty of mole fraction (10^4^U)
308	12	769	0.0814	0.0021	0.0643	0.0055
	15	817	0.1266	0.0042	0.1060	0.0098
	18	849	0.1327	0.0010	0.1156	0.0062
	21	875	0.1411	0.0051	0.1265	0.0118
	24	896	0.1501	0.0063	0.1378	0.0137
	27	914	0.1806	0.0071	0.1692	0.0161
318	12	661	0.0706	0.0023	0.0479	0.0052
	15	744	0.1182	0.0031	0.0901	0.0081
	18	791	0.1515	0.0032	0.1228	0.0091
	21	824	0.1601	0.0041	0.1353	0.0107
	24	851	0.2040	0.0064	0.1812	0.0151
	27	872	0.2079	0.0093	0.1858	0.0202
328	12	509	0.0611	0.0031	0.0319	0.0066
	15	656	0.1044	0.0023	0.0702	0.0062
	18	725	0.1620	0.0032	0.1203	0.0094
	21	769	0.1860	0.0042	0.1467	0.0115
	24	802	0.2248	0.0091	0.1849	0.0206
	27	829	0.2260	0.0021	0.1920	0.0107
338	12	388	0.0514	0.0023	0.0204	0.0047
	15	557	0.0928	0.0011	0.0530	0.0047
	18	652	0.2002	0.0101	0.1338	0.0219
	21	710	0.2266	0.0112	0.1650	0.0242
	24	751	0.2637	0.0103	0.2030	0.0231
	27	783	0.2590	0.0091	0.2079	0.0213

The experimental standard deviation was obtained by $S(y_{k} ) = \sqrt {\frac{{\sum\limits_{j = 1}^{n} {(y_{j} - \overline{y})^{2} } }}{n - 1}}$. Expanded uncertainty (U) = k*u_*combined*_ and the relative combined standard uncertainty $u_{combined}{/y} = \sqrt{ {{\sum\limits_{i = 1}^{N} {({P_{i}}u (x_i)} }}/{x_i})^2}$

^a^Standard uncertainty u are u(T) =  ± 0.1 K; u(p) =  ± 0.1 MPa. The value of the coverage factor k = 2 was chosen on the basis of the level of confidence of approximately 95 percent.

**Table 3:** Critical and physical properties of *empagliflozin* and CO_2_.

Substance	T_c_ (K)	P_c_(Pa)	$\omega$	V^s^ × 10^–6^ (m^3^/mol)	T(K)			
					P_sub_ (Pa)^e^			
					308	318	328	338
*Empagliflozin*	870.367^a^	18.7565^b^	0.479^c^	184.397^d^	0.0034	0.0089	0.022	0.0508
CO_2_	304.18	73.8	0.225					

T_c_: critical temperature; P_c_: critical pressure; $\omega$: acentric factor; V^s^: solid molar volume; T: temperature.

^a^Estimated by Fedors method.

^b^Estimated by the Joback and Reed method.

^c^Estimated by Lee-Kesler vapour pressure relations (the required normal boiling temperature (at 1.0 atm), T_b_ is estimated with Klincewicz relation, T_c_ = 50.2–0.16 M + 1.41 Tb, where M is molecular weight).

^d^Estimated by Immirzi, A., Perini, B method.

^e^Estimated by Lee-Kesler vapour method.

**Figure 5:**
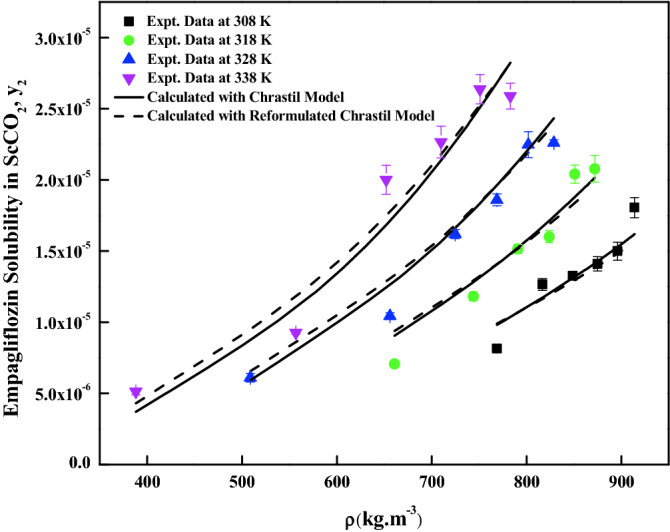
*Empagliflozin* solubility vs. ScCO_2_ density. Solid lines and broken lines are calculated solubilities with Chrastil and Reformulated Chrastil models, respectively.

**Figure 6:**
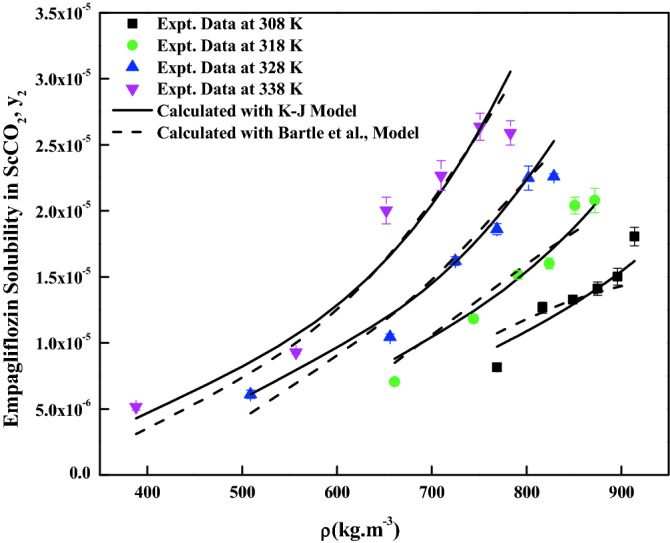
*Empagliflozin* solubility vs. ScCO_2_ density. Solid lines and broken lines are calculated solubilities with KJ and Bartle et al., models, respectively.

**Figure 7:**
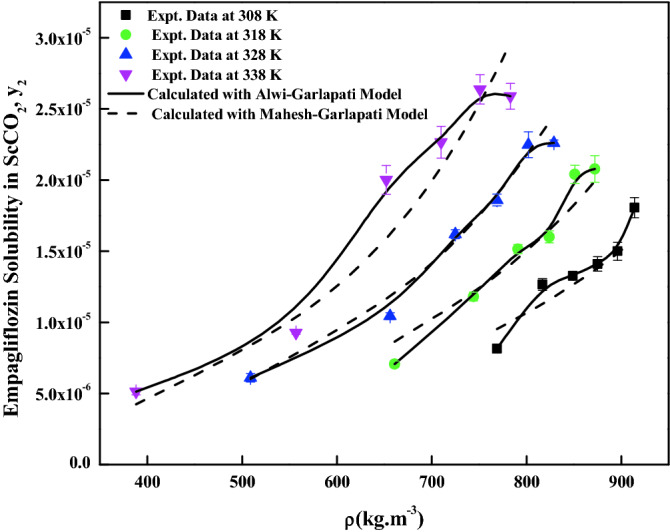
*Empagliflozin* solubility vs. ScCO_2_ density. Solid lines and broken lines are calculated solubilities with Alwi–Garlapati and Mahesh–Garlapati models, respectively.

**Figure 8:**
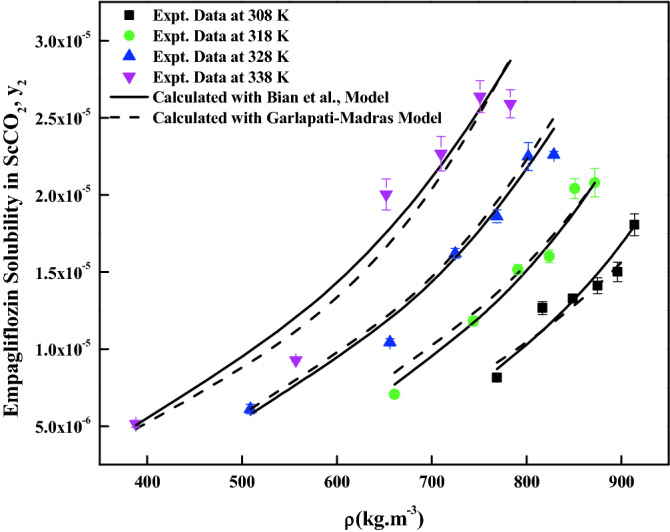
*Empagliflozin* solubility vs. ScCO_2_ density. Solid lines and broken lines are calculated solubilities with Bian et al., and Garlapati–Madras models, respectively.

**Figure 9:**
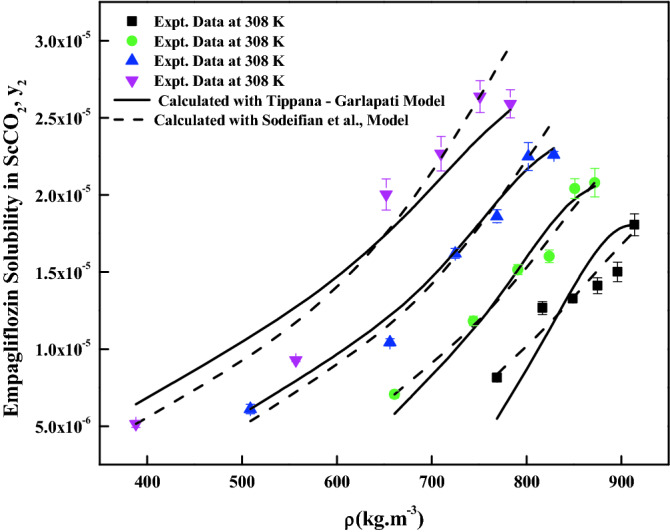
*Empagliflozin* solubility vs. ScCO_2_ density. Solid lines and broken lines are calculated solubilities with Tippana–Garlapati and Sodeifian et al., models, respectively.

**Figure 10:**
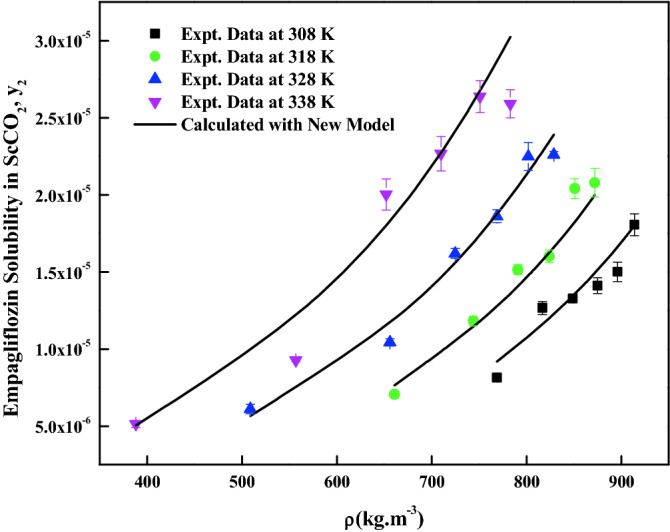
*Empagliflozin* solubility vs. ScCO_2_ density. Solid lines are calculated solubilities with new model.

**Figure 11:**
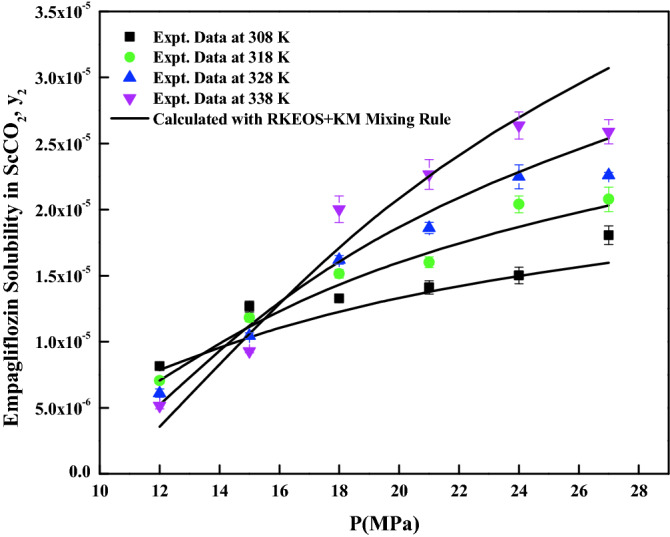
*Empagliflozin* solubility vs. pressure. Solid lines are calculated solubilities with RKEoS + KM mixing rule.

The density-based models considered in this work have different number of adjustable parameters. These parameters range from three to six numbers. The regression results of all the models are indicated in Tables 4 and 5. The correlating ability of the models is depicted in Figs. 5, 6, 7, 8, 9, 10, 11. From the results, it is clear that all the models are able to correlate the data reasonably well and maximum AARD% is observed to be 10.4%. It is believed that, more parameter models are able to correlate the data more accurately. Sodefian et al., model is able to correlate the data with AARD = 5.84% and Akaike’s information criterion (AIC = − 637.59) (more relevant information is presented in the following section). Among density models, Bian et al., model (five parameters model) is able to correlate the data well and corresponding AARD% is 5.1%. Interestingly, Chrastil (three parameters model) and Reformulated Chrastil models (three parameters model) are also able to correlate the data quite well. Further, Chrastil and Reformulated Chrastil models are able to provide the total enthalpy. Whereas, Bartle et al., model parameters are able to provide sublimation enthalpy of the *empagliflozin* drug. From the magnitude difference between the total and sublimation enthalpies, a solvation enthalpy is calculated. These results are reported in Table 6.

**Table 4:** Correlation constants for the exiting empirical models.

Name of the empirical model	Correlation parameters	AARD%	R^2^	R^2^_adj_
Alwi–Garlapati model	$A_{1}$ = − 1.8293; $B_{1}$ = − 14.218; $C_{1}$ = 2.8519	6.58	0.941	0.932
Bartle et al., model	$A_{2}$ = 12.195; $B_{2}$ = − 5972.3; $C_{2}$ = 7.7336 × 10^–3^	10.4	0.922	0.910
Bian et al., model	$A_{3}$ = − 0.062205; $B_{3}$ = − 5.7629 × 10^–4^; $C_{3}$ = − 6230.8; $D_{3}$ = 2.9473; $E_{3}$ = 4.5582	5.1	0.951	0.938
Chrastil model	$\kappa$ = 3.9083; $A_{4}$ = − 18.97; $B_{4}$ = − 3674.3	9.21	0.943	0.934
Garlapati–Madras model	$A_{5}$ = − 750.2; $B_{5}$ = 852.82; $C_{5}$ = 1.0855; $D_{5}$ = − 7397.7; $E_{5}$ = − 11.163	7.09	0.946	0.930
Kumar–Jonstone model	$A_{6}$ = − 14.274; $B_{6}$ = − 0.53652; $C_{6}$ = 2.1216	27.3	0.902	0.892
Mahesh_Garlapati model	$A_{7}$ = − 14.266; $B_{7}$ = − 0.52714; $C_{7}$ = 2.0972	8.14	0.931	0.921
Mendez–Teja model	$A_{8}$ = − 7775.4; $B_{8}$ = 2.3557; $C_{8}$ = 12.694	9.95	0.924	0.912
Sodefian et al., model	$A_{9}$ = − 23.94; $B_{9}$ = 1.6043 × 10^–3^; $C_{9}$ = 2.4939; $D_{9}$ = 2.6639 × 10^–4^; $E_{9}$ = − 9.5238 × 10^–3^; $F_{9}$ = − 1.037 × 10^3^	5.84	0.956	0.940
Reformulated Chrastil model	$\kappa^{\prime}$ = 3.8748; $A_{10}$ = − 33.58; $B_{10}$ = − 2705.8	9.14	0.943	0.935
Tippana–Garlapati model	$A_{11}$ = − 6.4027 × 10^–5^; $B_{11}$ = − 1.1813 × 10^–5^; $C_{11}$ = 2.0367 × 10^–5^; $D_{11}$ = 4.4544 × 10^–5^; $E_{11}$ = 3.5989 × 10^–5^; $F_{11}$ = − 2.4670 × 10^–5^	6.63	0.927	0.924

**Table 5:** Calculated result for the new model and RKEoS + Kwak-Mansoori mixing rule model.

Model	Correlation parameters	AARD%	R^2^	R^2^_adj_
New model	A = 36,634; B = − 0.096039; C = − 9673.6; D = − 0.16480	7.22	0.949	0.941
RKEoS + Kwak Mansoori mixing rule model	$k_{ij}$ = 0.32061; $l_{ij}$ = 0.1949	8.07	0.951	0.946

**Table 6:** Computed thermodynamic properties.

Model	Thermodynamic property		
	Total entalpy, ΔH_total_ (kJ/mol)	Enthalpy of sublimation, ΔH_sub_ (kJ/mol)	Enthalpy of solvation,$\Delta H_{sol}$ (kJ/mol)
Chrastil model	30.548^a^		− 19.105^d^
Reformulated Chrastil model	22.496^b^		− 27.157^e^
Bartle et al., model		49.653^c^ (approximate value)	

^a^Obtained with Chrastil model

^b^Obtained with Reformulated Chrastil model

^c^Obtained with Bartle et al.

^d^Obtained as a result of difference between the ΔH_sub_^c^ and ΔH_total_^a^.

^e^Obtained as a result between the ΔH_sub_^c^ and ΔH_total_^b^.

A new explicit solubility model based on solid–liquid equilibrium criteria combined with Wilson activity coefficient model corresponding to infinitesimal dilution is derived. The new model has four parameters $A$, $B$, $C$ and $D$. While regression, new model parameters are treated as temperature independent and solid molar volume is kept constant. The new model requires melting point, melting enthalpy and molar volume of *empagliflozin* drug, and these values are obtained from literature and group contribution methods. From literature^31^, the melting point of *empagliflozin* drug (426.1 K), molar volume (3.2699 × 10^–4^ m^3^/mol) and melting enthalpy (60.238 kJ/mol) are calculated based on literature, Immirzi and Perini^63^ and Jain et al., methods^66^, respectively. The new model makes use of objective function given in Eq. (33). Similarly, RKEoS along KMmr correlations are established with the help of critical properties given in Table 3 (temperature independent correlations). The optimization results of the new solubility and RKEoS models are indicated in Table 5.

In order to examine the ability of models for correlating the solubility data, AIC is applied^67–70^. When the data number is less than < 40, the corrected AIC (AIC_c_) is used.

35 $$ AIC_{c} = AIC + \frac{{2Q\left( {Q + 1} \right)}}{N - Q - 1} $$

where AIC, N, $Q$ and SSE are $N\;\ln \left( {{{SSE} / N}} \right) + 2Q$, the number of observations, the number of adjustable parameters of the model and the error sum of squares, respectively. According to AIC_c_ criterion, the best model has the least AIC_c_ value. Table 7 shows AIC_c_ values for various models considered in this study. In terms of AIC_c_, all the models are able to correlate the data closely. However, Reformulated Chrastil model has AIC_c_ value (− 637.02), hence it is treated as the best model and at the same time, Tippana–Garlapati model has the highest AIC_c_ value (− 621.69), therefore, it is considered as a poorly correlating model. Three parameters models namely Chrastil, Alwi–Garlapati and Mendez–Teja models have AIC_c_ values − 636.95, − 635.3 and − 635.4, respectively. The new model which has four parameters, indicating comparable performance with the best model (AIC_c_ value of − 637.24).

**Table 7:** Statistical quantities (SSE, RMSE, AIC and AIC_c_) of various models.

Model	SSE.10^11^	RMSE.10^6^	N	Q	AIC	AIC_c_
**Existing density models**						
Alwi–Garlapati model	5.673	1.537	3	24	− 636.5	− 635.30
Bartle et al., model	7.391	1.755	3	24	− 630.15	− 628.95
Bian et al., model	4.338	1.345	5	24	− 638.94	− 635.6
Chrastil model	5.297	1.486	3	24	− 638.15	− 636.95
Garlapati–Madras model	4.828	1.418	5	24	− 636.37	− 633.04
Kumar–Jonstone model	6.537	1.650	3	24	− 633.1	− 631.9
Mahesh_Garlapati model	6.337	1.625	3	24	− 633.84	− 632.64
Mendez–Teja model	5.650	7.51	3	24	− 636.60	− 635.4
Sodefian et al., model	4.222	1.326	6	24	− 637.59	− 632.65
Reformulated Chrastil model	5.280	1.483	3	24	− 638.22	− 637.02
Tippana–Garlapati model	6.659	1.666	6	24	− 627.0	− 621.69
**New model**						
New solid–liquid equilibrium model	4.635	1.389	4	24	− 639.35	− 637.24
**EoS model**						
RKEoS model + Kwak-Mansoori mixing rule	6.201	1.607	2	24	− 636.36	− 635.79

## Conclusion

Solubilities of *empagliflozin* in ScCO_2_ at temperatures (T = 308–338 K) and pressures (P = 12–27 MPa) were reported for the first time. The measured solubility in terms of mole faction ranged from 5.14 × 10^–6^ to 25.9 × 10^–6^. The data was successfully correlated with several models, Bian et al., model (AARD = 5.1%) was observed to be the best model in correlating the solubility data. All the models are able correlate the data reasonable. However, the correlating ability in ascending order of various models in terms of the lowest AIC_c_ values is as follows: Bian et al., Reformulated Chrastil, Chrastil, new solid–liquid equilibrium, Mendez–Teja, RKEoS + KMmr, Alwi–Garlapati, Sodefian et al., Mahesh–Garlapati, Bartle et al., Tippana–Garlapati models. The new model proposed in this work may be useful for correlating solids solubility in any SCF.

## Data Availability

The datasets generated and/or analysed during the current study are not publicly available due to confidential cases are available from the corresponding author on reasonable request.

## List of symbols

A-D: New model constants
$A_{1} - \;C_{1}$: Alwi–Garlapati model constants
$A_{2} - C_{2}$: Bartle model constants
$A_{3} - E_{3}$: Bian model constants
$A_{4} \;{\text{and}}\;B_{4}$: Chrastil model constants
$A_{5} - E_{5}$: Garlapati–Madras model constants
$A_{6} - C_{6}$: Kumar–Johnstone model constants
$A_{7} - C_{7}$: Mahesh–Garlapati model constants
$A_{8} - C_{8}$: Mendez–Teja model constants
$A_{9} - F_{9}$: Sodefian model constants
$A_{10} \;{\text{and}}\;B_{10}$: Reformulated Chrastil model constants
$A_{11} - F_{11}$: Tippana–Garlapati model constants
AARD: Absolute average relative deviation
Adj.R^2^: Adjusted R^2^
AIC: Akaike’s information criterion
a_ij_: EoS energy parameter
b_ij_: EoS volume correction
C: Solubility in Chrastil model
C_p_: Heat capacity
EoS: Equation of state
H_sol_: Solvation enthalpy
H_sub_: Sublimation enthalpy
H_Total_: Total enthalpy
H_2_^m^: Melting enthalpy of the solute
Mscf: Molecular weight of supercritical fluid
N: Number of data points
P: Total pressure
P^sub^: Sublimation pressure
RK: Redlich–Kwong
P_r_: Reduced pressure
P_c_: Critical pressure
Q: Number of parameters of a model
R: Universal gas constant
R^2^: Square of correlation coefficient
RMSE: Root mean square deviation
SSE: Error sum of squares
T: Temperature
T_c_: Critical temperature
T_m_: Melting temperature
Tr: Reduced temperature
y_2_: Solubility in molefraction

## Greek symbols

$$\Delta$$: Difference
$\hat{\varphi }_{i}^{S}$: Fugacity coefficient of the pure substance at saturation
$\hat{\varphi }_{i}^{{ScCO_{2} }}$: Solute fugacity in supercritical carbon dioxide (ScCO_2_)
$$\varpi$$: Acentric factor
$$\rho$$: Density
$$\rho_{r}$$: Reduced density
$\kappa_{ij}$: EoS mixing rule parameter
$l_{ij}$: EoS mixing rule parameter
$\lambda_{12} ,\lambda_{21}$: Wilson model parameters
$\gamma_{2}^{\infty }$: Infinite dilution activity coefficient

## Sub and superscripts

exp: Experimental
cal: Calculated
1: Solvent (CO_2_)
2: Solute (drug)
c: Critical
m: Melting
r: Reduced
